# Superior control of inflammatory pain by corticotropin-releasing factor receptor 1 via opioid peptides in distinct pain-relevant brain areas

**DOI:** 10.1186/s12974-022-02498-8

**Published:** 2022-06-15

**Authors:** Shaaban A. Mousa, Baled I. Khalefa, Mohammed Shaqura, Mohammed Al-Madol, Sascha Treskatsch, Michael Schäfer

**Affiliations:** 1 Department of Anesthesiology and Operative Intensive Care Medicine, Charité—Universitätsmedizin Berlin, Corporate Member of Freie Universität Berlin, Humboldt-Universität Zu Berlin, and Berlin Institute of Health, Charité Campus Benjamin Franklin, Hindenburgdamm 30, 12203 Berlin, Germany; 2 Zoology Department, Faculty of Science, AL-Zintan University, Alzintan, Libya

**Keywords:** Inflammatory pain, Corticotropin-releasing factor, Brain, Opioid peptide, Immunofluorescence

## Abstract

**Background:**

Under inflammatory conditions, the activation of corticotropin-releasing factor (CRF) receptor has been shown to inhibit pain through opioid peptide release from immune cells or neurons. CRF’s effects on human and animal pain modulation depend, however, on the distribution of its receptor subtypes 1 and 2 (CRF-R1 and CRF-R2) along the neuraxis of pain transmission. The objective of this study is to investigate the respective role of each CRF receptor subtype on centrally administered CRF-induced antinociception during inflammatory pain.

**Methods:**

The present study investigated the role of intracerebroventricular (i.c.v.) CRF receptor agonists on nociception and the contribution of cerebral CRF-R1 and/or CRF-R2 subtypes in an animal model of Freund’s complete adjuvant (FCA)-induced hind paw inflammation. Methods used included behavioral experiments, immunofluorescence confocal analysis, and reverse transcriptase-polymerase chain reaction.

**Results:**

Intracerebroventricular, but systemically inactive, doses of CRF elicited potent, dose-dependent antinociceptive effects in inflammatory pain which were significantly antagonized by i.c.v. CRF-R1-selective antagonist NBI 27914 (by approximately 60%) but less by CRF-R2-selective antagonist K41498 (by only 20%). In line with these findings, i.c.v. administration of CRF-R1 agonist stressin I produced superior control of inflammatory pain over CRF-R2 agonist urocortin-2. Intriguingly, i.c.v. opioid antagonist naloxone significantly reversed the CRF as well as CRF-R1 agonist-elicited pain inhibition. Consistent with existing evidence of high CRF concentrations in brain areas such as the thalamus, hypothalamus, locus coeruleus, and periaqueductal gray following its i.c.v. administration, double-immunofluorescence confocal microscopy demonstrated primarily CRF-R1-positive neurons that expressed opioid peptides in these pain-relevant brain areas. Finally, PCR analysis confirmed the predominant expression of the CRF-R1 over CRF-R2 in representative brain areas such as the hypothalamus.

**Conclusion:**

Taken together, these findings suggest that CRF-R1 in opioid-peptide-containing brain areas plays an important role in the modulation of inflammatory pain and may be a useful therapeutic target for inflammatory pain control.

**Supplementary Information:**

The online version contains supplementary material available at 10.1186/s12974-022-02498-8.

## Introduction

CRF plays a crucial role in the stress response by modulating behavioral and neuroendocrine responses [1] one of which is the modulation of pain [2]. Indeed, CRF injected into the rat brain has been reported to modulate both visceral and somatic pain [2–7]. In one of our previous studies, we found that i.c.v. application of CRF, a non-selective CRF-R ligand, inhibited hyperalgesia after Freund’s complete adjuvant (FCA)-induced rat hindpaw inflammation through opioid peptides [4].

Opioid peptides are expressed throughout the central nervous system [8] predominantly in the brain [7, 9, 10] and spinal cord areas [11–13] that are well established as modulators of incoming painful stimuli. During painful inflammatory states they are reported to be up-regulated and to contribute to the modulation of pain [4, 10, 11, 13]. For example, within the rostral ventromedial medulla of the brain, opto-/chemogenic inhibition of enkephalinergic/GABAergic interneurons resulted in an enhanced facilitation of mechanical pain [12]. Moreover, within the dorsal horn of the spinal cord, the opioid peptide enkephalin is expressed in a specific population of spinal inhibitory interneurons that regulate the transmission of painful stimuli [13]. Intriguingly, recent studies demonstrated that these enkephalinergic inhibitory interneurons also express CRF receptors [4], particularly the CRF-R2 receptor [14], and are involved in inflammatory pain inhibition.

The role of CRF receptors in nociceptive transmission is complex: Their activation could inhibit or facilitate nociceptive transmission in experimental animals depending on the distribution of CRF-R1 and CRF-R2 in the nervous system. A recent study [15] found that CRF-R1 knock-out mice with latent sensitization induced by a hind paw incision and remifentanil application revealed stronger hyperalgesic responses to naloxone than wild-type mice. Consistently, i.c.v. CRF increased visceromotor responses indicating visceral hypersensitivity [16, 17] which was blocked by a centrally administered non-selective CRF receptor antagonist [17] or by a systemically administered CRF-R1 selective antagonist [16]. Despite the evidence that the central CRF system plays a role in pain modulation, particularly through endogenous opioid peptides [2, 4], it is not clear which brain CRF receptor subtype is predominant in mediating the control of tonic inflammatory pain.

Therefore, this study extends our previous investigations [4] now using highly selective agonists and antagonists for CRF-R1 and CRF-R2 in order to determine the respective role of each receptor subtype on centrally administered CRF-induced analgesia under inflammatory conditions. In an animal model of tonic rat’s hindpaw inflammation, we examined the involvement of the brain CRF-R1 and CRF-R2 in the effects of i.c.v. administered CRF using the selective CRF-R1 (NBI 27914) and CRF-R2 (K41498) antagonists. For further confirmation, the antinociceptive effects of i.c.v.-applied CRF-R1 (stressin I) and CRF-R2 (urocortin-2) selective agonists, alone or in combination with their corresponding selective antagonists (NBI 27914 for CRF-R1 and K41498 for CRF-R2, respectively), were assessed following mechanical painful stimuli. In addition, we examined whether antinociceptive effects of i.c.v. CRF and stressin I can be attenuated by i.c.v. opioid-receptor antagonist naloxone. Finally, we investigated the expression of CRF-R1 and CRF-R2 and its co-localization with opioid peptides in pain-relevant brain areas by employing double-immunofluorescence microscopy.

## Materials and methods

### Animals

In order to avoid potential interferences of menstrual and hormonal fluctuations with our behavioral testing and for better comparisons with previous studies that were done in male rats only, we conducted our experiments in male Wistar rats (200–250 g) (Charité-Universitätsmedizin Berlin, Campus Benjamin Franklin, Berlin, Germany). Rats were housed individually in cages and maintained on a 12 h light/dark schedule with food pellets and water ad libitum. Room temperature was maintained at 22 ± 0.5 °C and at a relative humidity between 60 and 65%. Experiments and animal care were performed according to the European guidelines and approved by the local animal care committee of the Senate of Berlin, Germany (Landesamt für Arbeitsschutz, Gesundheitsschutz und Technische Sicherheit, Berlin). All efforts were made to minimize the number of animals used and their suffering.

### Induction of inflammation

Rats received an intraplantar (i.pl.) injection of 0.15 ml FCA into the right hind paw under brief isoflurane (Willy Rüsch GmbH, Böblingen, Germany) anesthesia. This treatment consistently produces a unilateral local inflammation of the inoculated paw as reflected with an increase in paw volume, temperature and infiltration with various types of immune cells as previously described by Rittner et al. [18].

### Surgery to implant i.c.v. cannula

The i.c.v. cannulation surgery was performed as described elsewhere [4, 19, 20]. Rats were acclimated to the test situation for 3 days before cannulation. Under continuous isoflurane anesthesia via a loose-fitting plastic mask, rats were placed in a stereotactic apparatus and the skull was exposed. A burr hole was drilled above the location of the right lateral ventricle (coordinates: AP 0.25 mm, lateral 1.6 mm, ventral 4.0 mm related to bregma) [21]. A stainless-steel cannula guide pedestal was fixed to the skull over the burr hole for subsequent i.c.v. applications using two stainless-steel screws. Then, the entire assembly was held in place with dental cement. The cannula guide extended into the burr hole 1 mm below the pedestal but did not touch the surface of the cortex. After surgery, a stainless-steel blocker was inserted into the i.c.v. cannula. At least 3 days were allowed for recovery from surgery before subsequent i.c.v. injections and behavioral testing was performed. At the end of experiments, the correct cannula placements were verified by infusion of (1%) methylene blue dye inside the ventricular system. Drugs were injected in a volume of 10 µl followed by 10 µl of vehicle to flush the catheter.

### Drugs

The following drugs were used: rat/human CRF (Sigma-Aldrich, St. Louis, MO); CRF-R1 agonist stressin I and CRF-R1 antagonist NBI 27914 (Bio-Techne GmbH, Wiesbaden-Nordenstadt, Germany); CRF-R2 agonist urocortin-2 (Ucn-2), CRF-R2 antagonist K41498 (Bio-Techne GmbH, Wiesbaden-Nordenstadt, Germany); naloxone hydrochloride (Sigma-Aldrich, St. Louis, MO). Doses were calculated as the free base and drugs were dissolved in isotonic saline as vehicle. The volume of i.c.v. drug administration was 10 µl. For each dose a separate group of animals (*n* = 6) was used. Drugs were administered under brief isoflurane anesthesia.

### Algesiometric testing

Nociceptive thresholds were assessed by using the paw pressure test (modified Randall–Selitto test). Animals (*n* = 6 per group) were gently restrained under paper wadding and incremental pressure was applied via a wedge-shaped, blunt piston onto the dorsal surface of the hind paw by means of an automated gauge (Ugo Basile). The pressure required to elicit paw withdrawal, the paw pressure threshold (PPT), was determined. A cutoff of 250 g was used. Three consecutive trials, separated by intervals of 10 s, were conducted and the average was determined. Baseline PPT were tested before and 4 days after inoculation with FCA. The same procedure was performed on the contralateral side; the sequence of sides was alternated between subjects to preclude order effects. In all behavioral experiments, drugs were prepared by a different person (M. Sh.) and the examiner (B.K.) was unaware of the treatment that each animal received by chance.

### Receptor specificity

The most effective dose of i.c.v. CRF (2 µmol) was administered together with different doses of CRF-R1 antagonist NBI 27914 (0, 4, 6, 10 µmol) or CRF-R2 antagonist K41498 (0, 5, 8, 14 µmol) to determine the receptor specificity of CRF-mediated antinociceptive effects. The highest dose (2 µmol) of i.c.v. CRF used in this study has previously been shown to be systemically ineffective [4]. Next, the most effective doses of i.c.v. CRF-R1 agonist stressin I (2.2 µmol) or CRF-R2 agonist urocortin-2 (0.75 µmol) were administered alone or together with different doses of corresponding CRF-R1 antagonist NBI 27914 (0, 4, 8, 10 µmol) or CRF-R2 antagonist K41498 (0, 3, 5, 7.5, 10 µmol), respectively. The goal was to determine the receptor specificity of each CRF agonist-mediated antinociceptive effect. Finally, the most effective doses of i.c.v. CRF (2.0 µmol) or stressin I (2.2 µmol) were administered alone or together with different doses of naloxone.

### Tissue preparation

Four days after FCA inoculation, rats were deeply anesthetized with halothane and transcardially perfused with 100 ml warm saline, followed by 300 ml 4% (w/v) paraformaldehyde in 0.16 M phosphate buffer solution (pH 7.4). After perfusion, brain was removed, postfixed in the same fixatives for 90 min, and then cryoprotected overnight at 4 °C in PBS containing 10% sucrose. The tissues were embedded in tissue-Tek compound (OCT, Miles Inc. Elkhart, IN) and frozen, then serially cut at 40 µm on cryostat. Every fourth section of brain was collected in PBS (floating sections).

### Immunofluorescence staining

For single or double-immunofluorescence, tissue sections were processed as described previously [4, 14]. Coronal or parasagittal brain sections were incubated with primary antibodies overnight at 4 °C. The antibodies used included the rabbit polyclonal antibody CRF-R1 raised against a synthetic 17 amino acid peptide from N-terminus extracellular domain of CRF-R1 (MBL, Wobum, MA, USA; MC-1778) or the antibody CRF-R2 raised against a synthetic peptide corresponding to the extracellular N-terminal domain of CRF-R2 (Sigma; St Louis, MO, USA, C4241). These were applied alone or in combination with mouse monoclonal antibodies against POMC, vasopressin (1:1000, Chemicon International, MA) or panopioid 3E7 against the amino terminal H-Tyr-Gly-Gly-Phe sequence of ß-endorphin, which also has a high cross-reactivity with homologs of identical sequence such as ENK (10 μg/ml; Gramsch Laboratories, Schwabhausen, Germany). After incubation with primary antibodies, the tissue sections were washed with PBS and then incubated with Texas-Red-conjugated goat anti-rabbit antibody (Vector Laboratories) in combination with Alexa-Fluor-488 goat anti-mouse antibody (Invitrogen, Germany). Thereafter, sections were washed with PBS and the nuclei stained bright blue with 4'-6-diamidino-2-phenylindole (DAPI) (0.1 µg/ml in PBS) (Sigma). Finally, the tissues were washed in PBS, mounted in vectashield (Vector Laboratories) and imaged on a confocal laser scanning microscope, LSM510 (Carl Zeiss, Göttingen, Germany). To demonstrate specificity of staining, the following controls were included as mentioned in detail elsewhere [4, 22–24]: (i) omission of either the primary antisera or the secondary antibodies.

### CRF-R1 and CRF-R2 mRNA detection by conventional and quantitative RT-PCR

PCR analysis for CRF-R1 and CRF-R2 specific mRNA from rat hypothalamus was performed as described previously (for details see Li et al.)[25]. Total RNA was extracted from the hypothalamus of Wistar rats (*n* = 5 per experimental group) using RNeasy Kit (Qiagen, Hilden, Germany). 0.5 µl (25 pmol) oligo dT and 2 µl (200 pmol) random primers were added up to 1 μg total RNA, incubated at 37 °C for 15 min, then at 85 °C for 5 s, finally at 4 °C for transfer onto ice (according to TaKaRa® manual). cDNA was stored at − 20 °C. The following specific primers were used: for CRF-R1, forward primer: ACACTACCATGTTGCAGTC, reverse primer: GAACATCCAGAAGAAGTTGG (Ensembl, Accession Nr: NM_030999); for CRF-R2, forward primer: CACACTGTGAACCCATTT TGG, reverse primer: GATGAGTTGCAGCAGG (Ensembl, Accession NM_022714). Conventional PCR was performed with a Maxima Hotstart Green Enzyme kit (Thermo Fisher Scientific GmbH Berlin, Germany). Amplification was performed by Eppendorf PCR-Cycler Vapo.Protect **(**Eppendorf Vertrieb Deutschland GmbH, Wesseling-Berzdorf) out for 40 cycles**,** each consisting of 30 s at 95 °C and of 30 s at 60 °C and 30 s at 72 °C. Specific bands were visualized on 2% agarose gel plus 0.01% ethidium bromide; the entire PCR product of 20 μl migrated for 40 min at 100 V in the BioRad chamber system with 1 × TAE buffer. The imaging was obtained by using Gel Doc EZ Imager (Bio-Rad Laboratories GmbH, Feldkirchen, Germany).

Taqman® quantitative RT-PCR was performed with a SYBR® Green kit following the manufacturer’s instructions (Applied Biosystems). The following specific primers were used: for CRF-R1, forward primer: ACAGCCATTGTGCTCACGTA, reverse primer: ATGGGGAAAGGTACACCCCA (Ensembl, Accession Nr: NM_030999.4); for CRF-R2, forward primer: TACAACACGACCCGGAATGC, reverse primer: CTGCTTGTCATCCAAA ATGGGT (Ensembl, Accession NM_022714). Amplification was carried out for 40 cycles, each consisting of 15 s at 95 °C; for CRF-R1 and CRF-R2 specific mRNA and 18S ribosomal protein for 60 s at 60 °C. A temperature just below the specific melting temperature (Tm) was employed for detection of fluorescence specific products for CRF-R1 and CRF-R2 specific mRNA was quantified using three independent samples in duplicate. The housekeeping gene S18, a gene for a ribosomal protein, was used as an internal reference gene for quantification.

### Analysis of data

Data were analyzed using one-way ANOVA for multiple comparisons followed by Dunnett’s post hoc test for comparisons with controls. Quantitative RT-PCR data were analyzed as two group comparisons (CRF-R1 versus CRF-R2) by two-tailed independent Student t-test in case of normally distributed data. Differences were considered significant if *p* < 0.05. All tests were performed using using Sigma Plot 13.0 statistical software (Systat Software GmbH, Erkrath, Germany). Data are expressed as means ± S.D.

## Results

### Distinct antinociceptive effects of intracerebroventricular CRF-R1 and CRF-R2 receptor agonists in an animal model of inflammatory pain

**Fig. 1:**
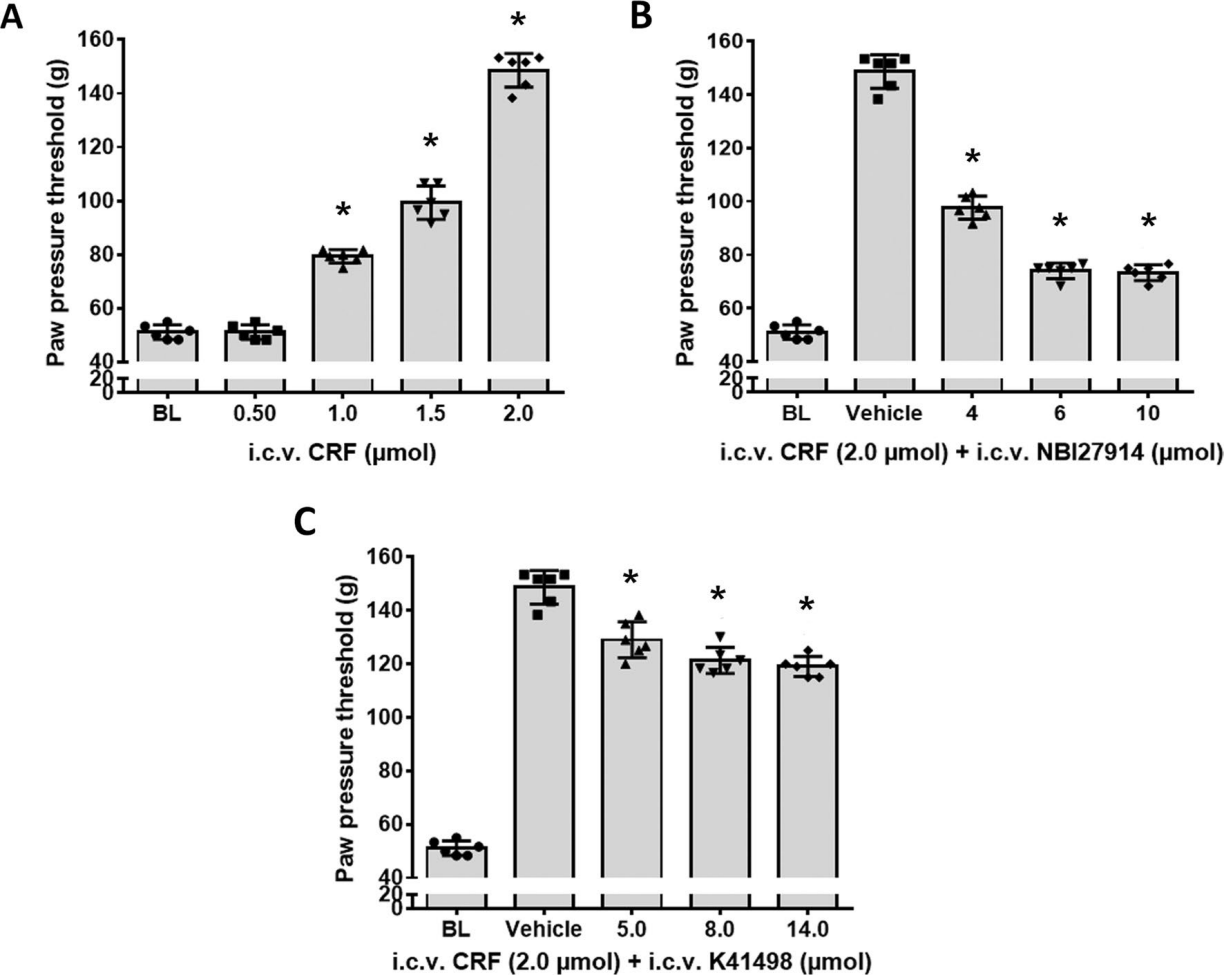
Antagonism of antinociceptive effects of i.c.v. administered CRF by CRF-R1 antagonist NBI 27914 and CRF-R2 antagonist K41498. In Wistar rats with 4-day FCA-induced hind paw inflammation, effects of i.c.v. administered CRF on nociceptive paw pressure thresholds were measured by algesiometry. **A** i.c.v. application of CRF (0.5, 01.0, 1.5, 2.0 µmol) significantly increased nociceptive thresholds in a dose-dependent manner (*F*_(4, 25)_ = 445; *P* < 0.001). **B** Dose-dependent antagonism of i.c.v. CRF’s antinociception by co-administered CRF-R1 antagonist NBI 27914 (*F*_(4, 25)_ = 502.9; *P* < 0.001). **C** In contrast, increasing doses of the i.c.v. CRF-R2 antagonist K41498 only partially antagonized CRF’s antinociception (*F*_(4, 25)_ = 315.7; *P* < 0.001). *indicates significant differences from vehicle treatment; data points (n = 6) represent means ± SD

In Wistar rats with hindpaw inflammation-induced mechanical hyperalgesia, i.c.v. application of CRF (0.5, 1.0, 1.5, 2 µmol) produced significant and dose-dependent increases of paw pressure thresholds in inflamed hindpaws (*F*_(4,25)_ = 445; *P* < 0.001) (Fig. 1A). This antinociceptive effect of i.c.v. CRF was greatly reversed (by approximately 60%) with increasing i.c.v. doses of the CRF-R1 selective antagonist NBI 27914 (*F*_(4, 25)_ = 502.9; *P* < 0.001) (Fig. 1B) but to a much lesser degree (only 20%) by the i.c.v. applied CRF-R2 selective antagonist K41498 (*F*_(4, 25)_ = 315.7; *P* < 0.001) (Fig. 1C). The highest dose (2 µmol) of i.c.v. CRF has previously been shown to be systemically ineffective [4].

**Fig. 2:**
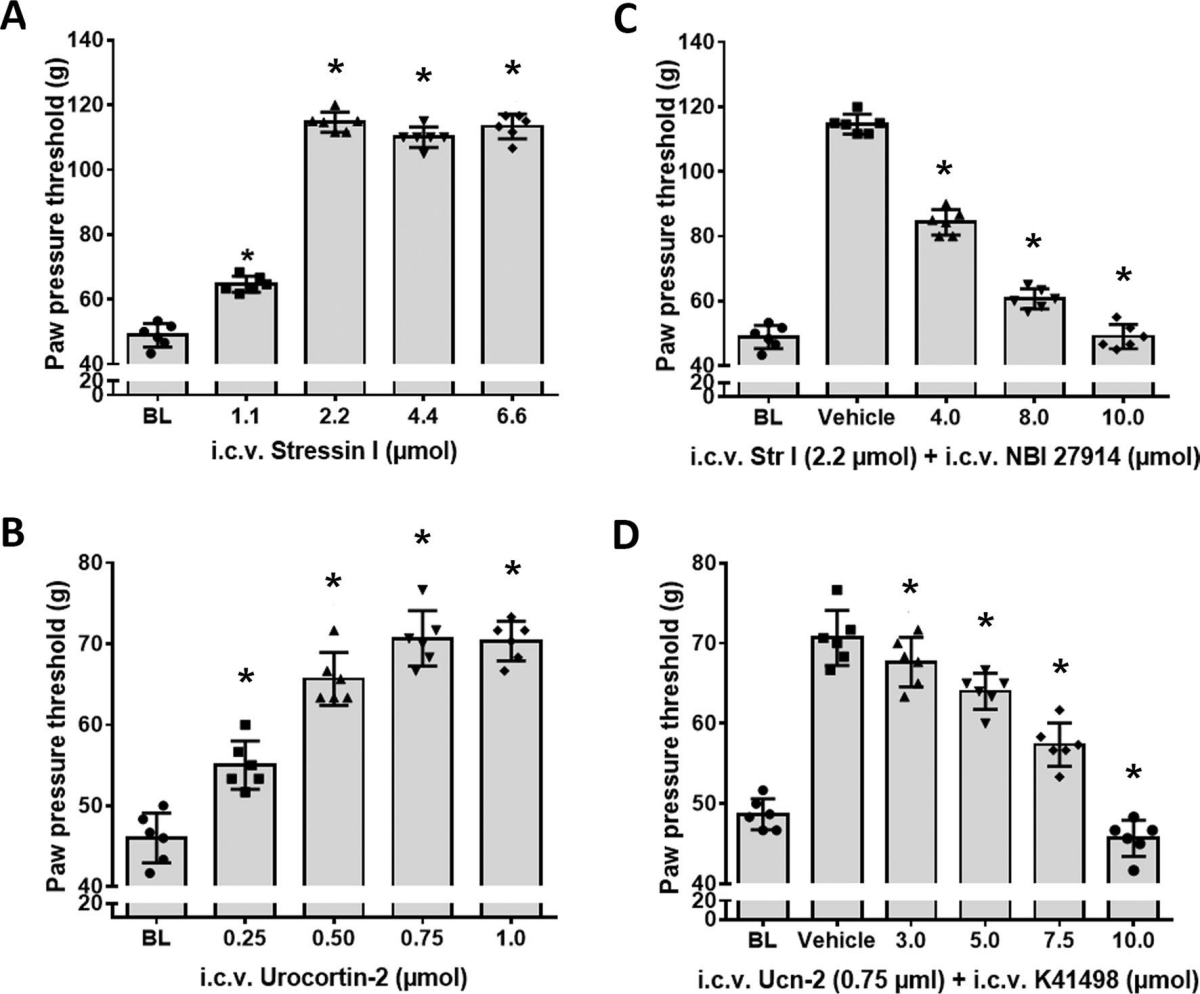
Antinociceptive effects of the i.c.v. CRF-R1 agonist stressin I or CRF-R2 agonist Ucn-2 and their antagonism by the respective CRF-R1 (NBI 27914) or CRF-R2 (K41498) selective antagonists. The effects of i.c.v. CRF-R1 (stressin I) or CRF-R2 (Ucn-2) agonists on nociceptive paw pressure thresholds were measured by algesiometry. **A** i.c.v. administration of the CRF-R1 agonist stressin I significantly increased nociceptive thresholds in a dose-dependent manner (*F*_(4, 25)_ = 552.4; *P* < 0.001). **B** i.c.v. administration of the CRF-R2 agonist Ucn-2 significantly increased nociceptive thresholds (*F*_(4, 25)_ = 389.1; *P* < 0.001). **C** Dose-dependent antagonism of i.c.v. CRF-R1 agonist’s antinociception by co-administered CRF-R1 antagonist NBI 27914 (*F*_(4, 25)_ = 73.9; *P* < 0.001, one-way ANOVA and Dunnett’s test). **D**) Dose-dependent antagonism of i.c.v. Ucn-2 antinociception by co-administered CRF-R2 antagonist K41498 was significant (*F*_(5, 30)_ = 88.4; *P* < 0.001), *indicates significant differences from vehicle treatment; data points (n = 6) represent means ± SD

**Fig. 3:**
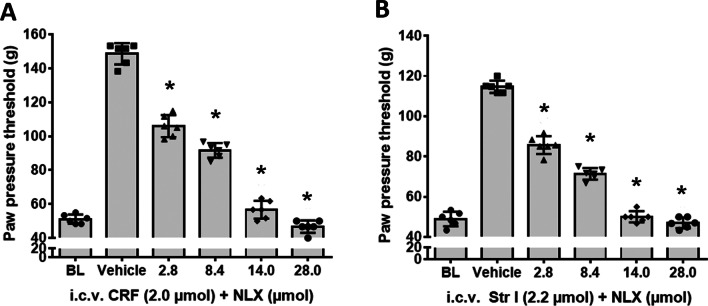
The antinociceptive effects of i.c.v. CRF and CRF-R1 agonist stressin I and their antagonism by the opioid receptor antagonist naloxone in rats with inflamed hindpaws. The effects of i.c.v. co-administration of the opioid receptor antagonist naloxone with CRF or CRF-R1 agonist stressin I on nociceptive thresholds were measured by algesiometer. **A** The i.c.v. CRF’s induced-antinociception was significantly reduced by co-administration of the opioid receptor antagonist naloxone (*F*_(5, 30)_ = 384.8; *P* < 0.001). **B** Similarly, the antinociception resulting from i.c.v. CRF-R1 agonist stressin I was significantly attenuated by co-administered opioid receptor antagonist naloxone (*F*_(5, 30)_ = 400.8; *P* < 0.001) *indicates significant differences from vehicle treatment); data points (*n* = 6) represent means ± SD

Corroborating these findings, i.c.v. administration of the CRF-R1 selective agonist stressin I resulted in significant and dose-dependent increases of paw pressure thresholds (*F*_(4, 25)_ = 552.4; *P* < 0.001) (Fig. 2A) that were superior over the elevations of the i.c.v. CRF-R2 selective agonist Ucn-2 (*F*_(4, 25)_ = 389.1; *P* < 0.001) (Fig. 2B). Importantly, these antinociceptive effects of i.c.v. CRF-R1 selective agonist stressin I or CRF-R2 selective agonist Ucn-2 were antagonized by increasing i.c.v. doses of their corresponding selective antagonists NBI27914 or K41498, respectively (Fig. 2C; *F*_(4, 25)_ = 73.9 or Fig. 2D; *F*_(5, 30)_ = 88.4; *P* < 0.001). Interestingly, i.c.v. co-injection of either CRF (Fig. 3A) or stressin I (Fig. 3B) together with the opioid receptor antagonist naloxone dose-dependently reduced the antinociceptive effects of these substances (Fig. 3A; *F*_(5, 30)_ = 384.8 or Fig. 3B; *F*_(5, 30)_ = 400.8; *P* < 0.001), thus indicating an opioid receptor-mediated effect.

### Co-expression of CRF-R1 and CRF-R2 with opioid peptides in distinct pain-relevant brain areas

**Fig. 4:**
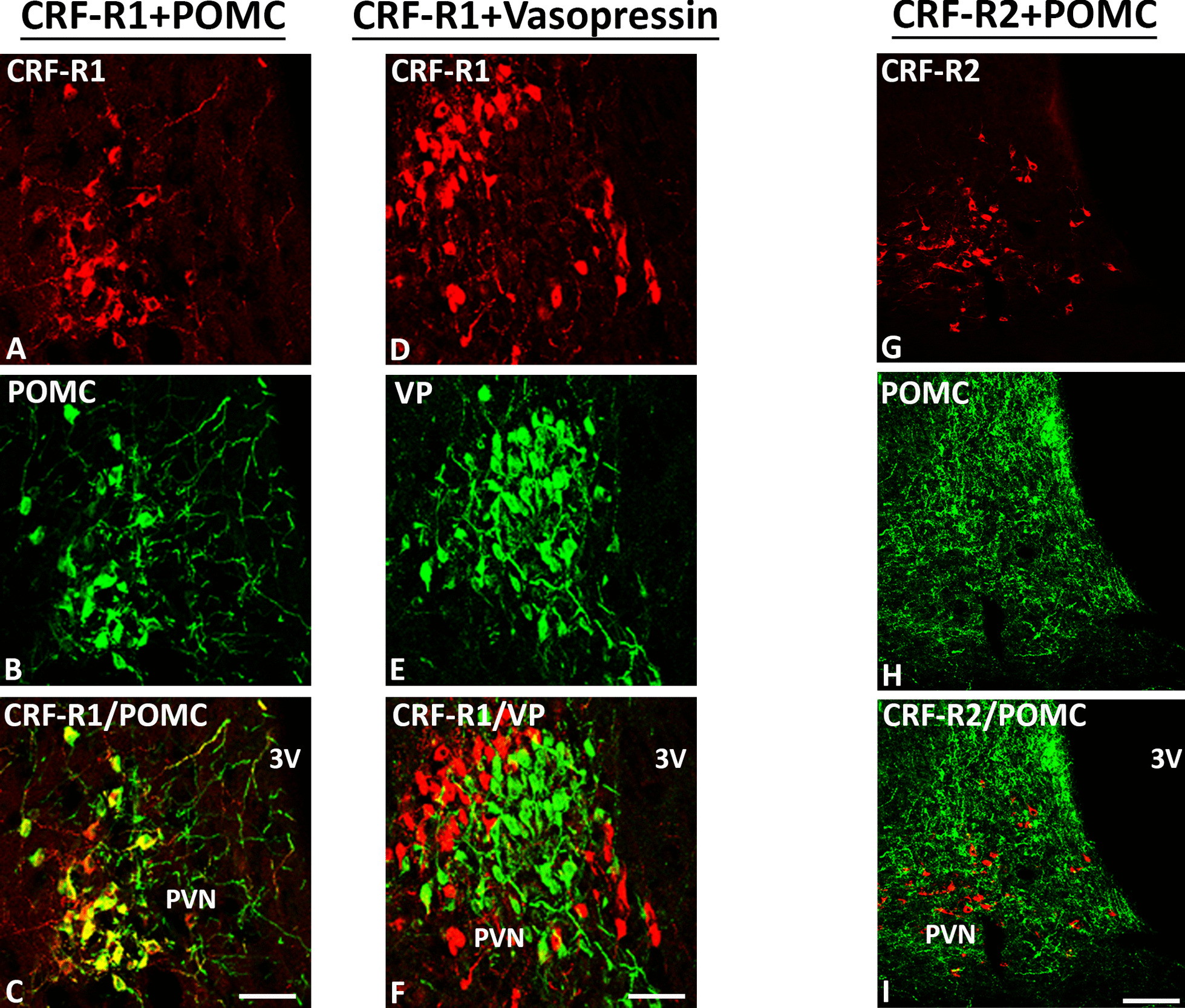
Double-immunofluorescence staining of CRF-R1 (*red fluorescence*) **A**–**F** or CRF-R2 **G**–**I** (*red fluorescence*) with proopiomelanocortin (POMC) (*green fluorescence*) **B, H** and vasopressin (*green fluorescence*) **E** in the paraventricular nucleus (PVN) of the rat hypothalamus. **A**–**C** Double-immunofluorescence staining of coronal brain sections of the rat with hind paw inflammation showing that CRF-R1-immunoreactive neurons within PVN overlap with the opioid peptide precursor POMC (**A**–**C**). Some neurons express CRF-R1 or POMC only. **D**–**F** Show CRF-R1-immunoreactive neurons in the paraventricular nucleus (PVN) residing in close vicinity of vasopressin positive cells without any overlap of the two cell groups. **G**–**I** Show only few scattered CRF-R2-immunoreactive neurons without POMC overlap in the same region. Bar = 20 μm

**Fig. 5:**
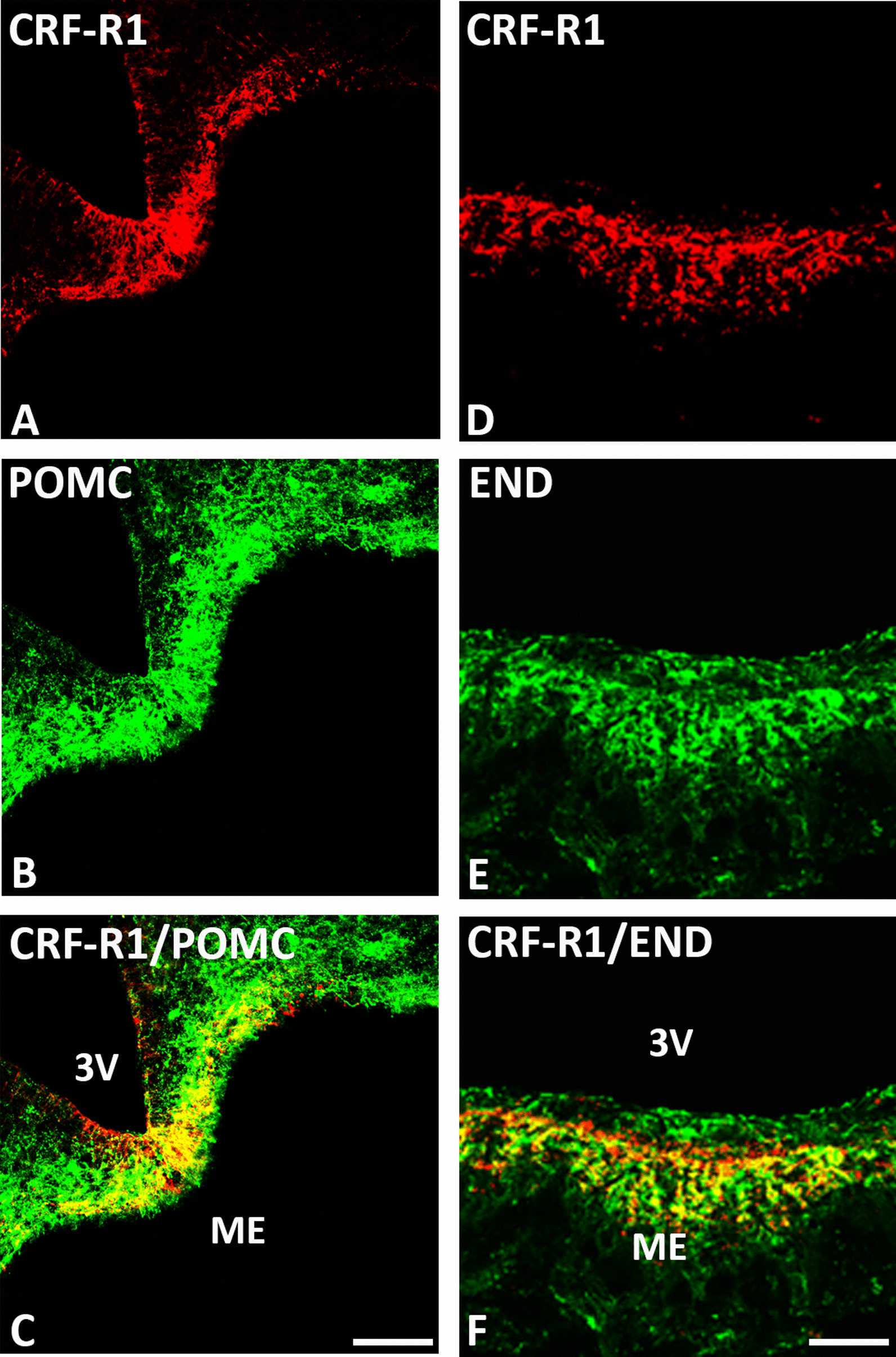
Double-immunofluorescence staining of CRF-R1 (*red fluorescence*) **A**–**F** and POMC (*green fluorescence*) **B**, **C** or β-endorphin (END) (*green fluorescence*) **E**, **F** in the median eminence of the rat hypothalamus.** A**–**F** Show that most of CRF-R1-immunoreactive fibers express POMC (**C**) or END (**F**) in coronal sections of the rat brain of Wistar rats, but few fibers contain CRF-R1, POMC or END only. Bar = 20 µm

**Fig. 6:**
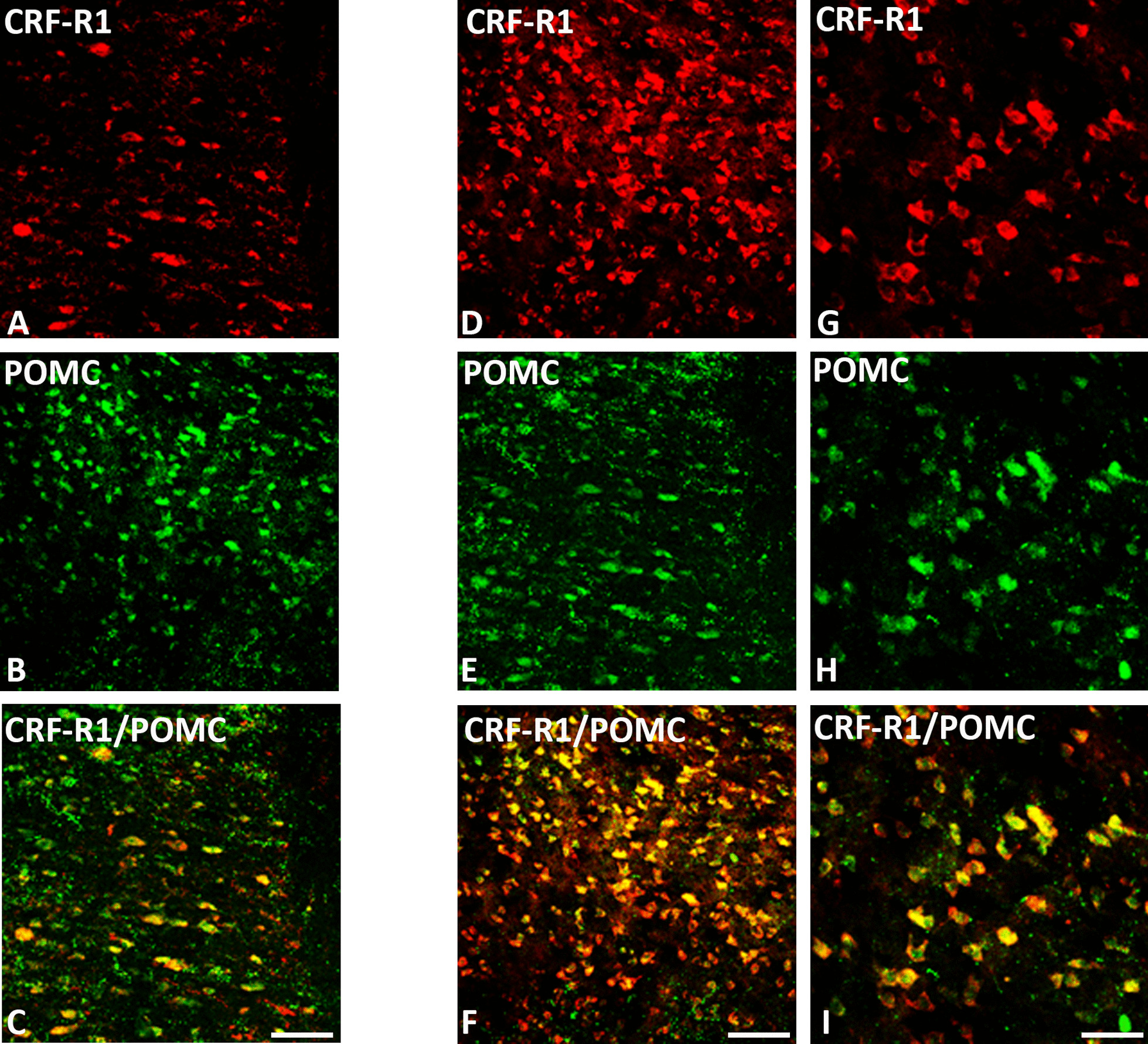
Double-immunofluorescence staining of CRF-R1 (*red fluorescence*) **A**, **D**, **G** and POMC (*green fluorescence*) **B**, **E**, **H** (*green fluorescence*) **E** in the thalamus **A**–**C** and periaqueductal grey **D**–**I** of rat brain. **A**–**C** Coronal sections of the rat brain with hindpaw inflammation show that most of CRF-R1-positive neurons in the thalamus region express POMC, but few fibers contain CRF-R1 or POMC only. **D**–**F** Coronal sections of the rat brain with hindpaw inflammation show co-localization of CRF-R1 with POMC neurons in the periaqueductal grey, but few fibers contain CRF-R1 or POMC only. Bar = 20 µm, **G**–**I** Higher magnification of **D**–**F**. Bar = 40 µm

**Fig. 7:**
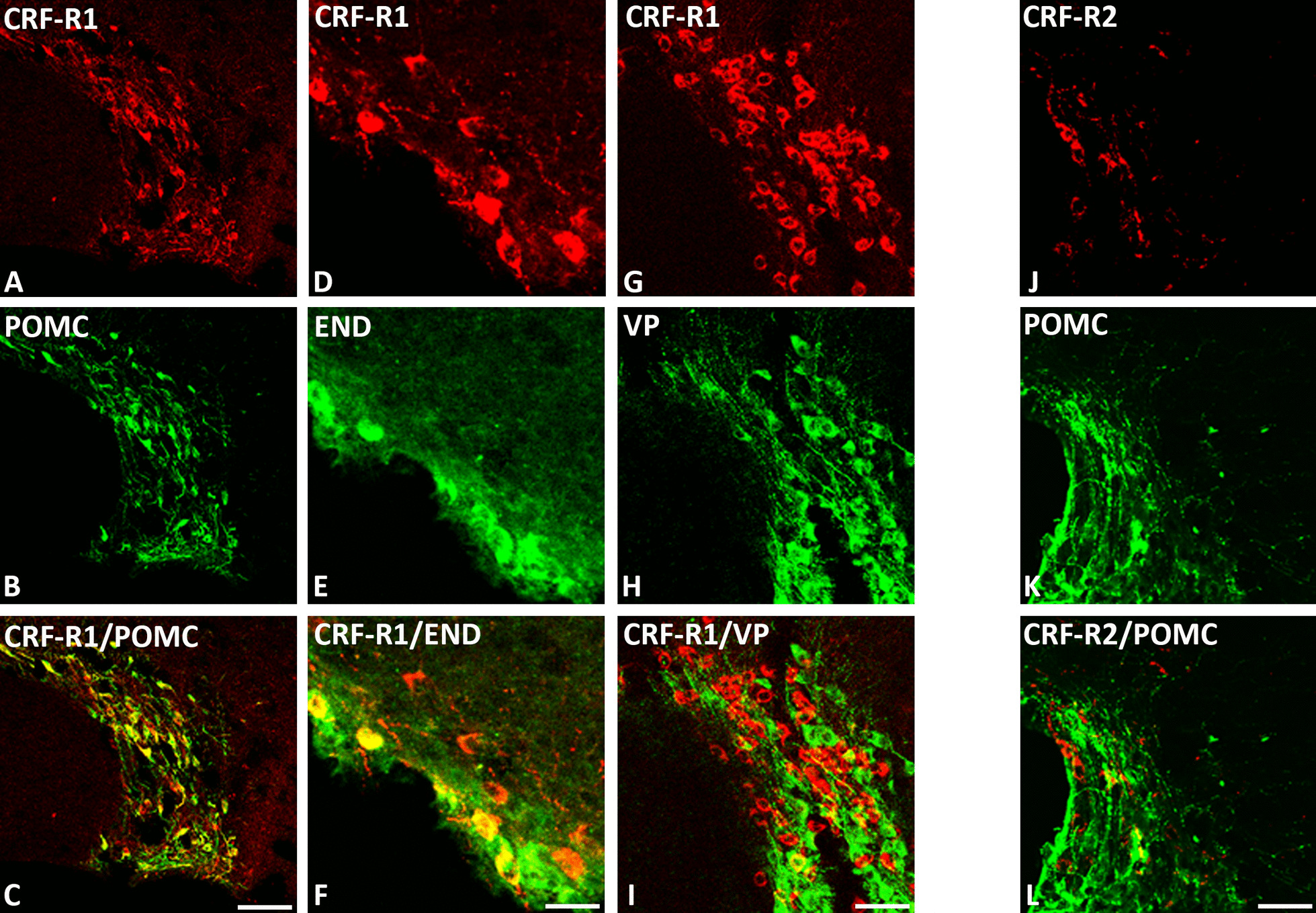
Double-immunofluorescence staining of CRF-R1 (*red fluorescence*) **A**, **D**, **G** or CRF-R2 **J**–**l** with proopiomelanocortin (POMC) (**B**, **K**), β-endorphin (END) **E** and vasopressin **H** (*green fluorescence*) in the supraoptic area (SOA) of the rat hypothalamus. **A**–**F** Double-immunofluorescence staining of coronal sections of the rat brain with hind paw inflammation showing that CRF-R-immunoreactive neurons within SON overlap with the opioid peptide precursor POMC **C** or (**F**). Some neurons express CRF-R1 or POMC only. **G**–**I** Show the majority of CRF-R1-immunoreactive neurons residing in close vicinity, and rarely overlap (as indicated by yellow fluorescence) with vasopressin positive cells. **J**–**L** Show few scattered CRF-R2-immunoreactive neurons with only rare overlap with POMC-ir neurons. Bar = 20 μm

**Fig. 8:**
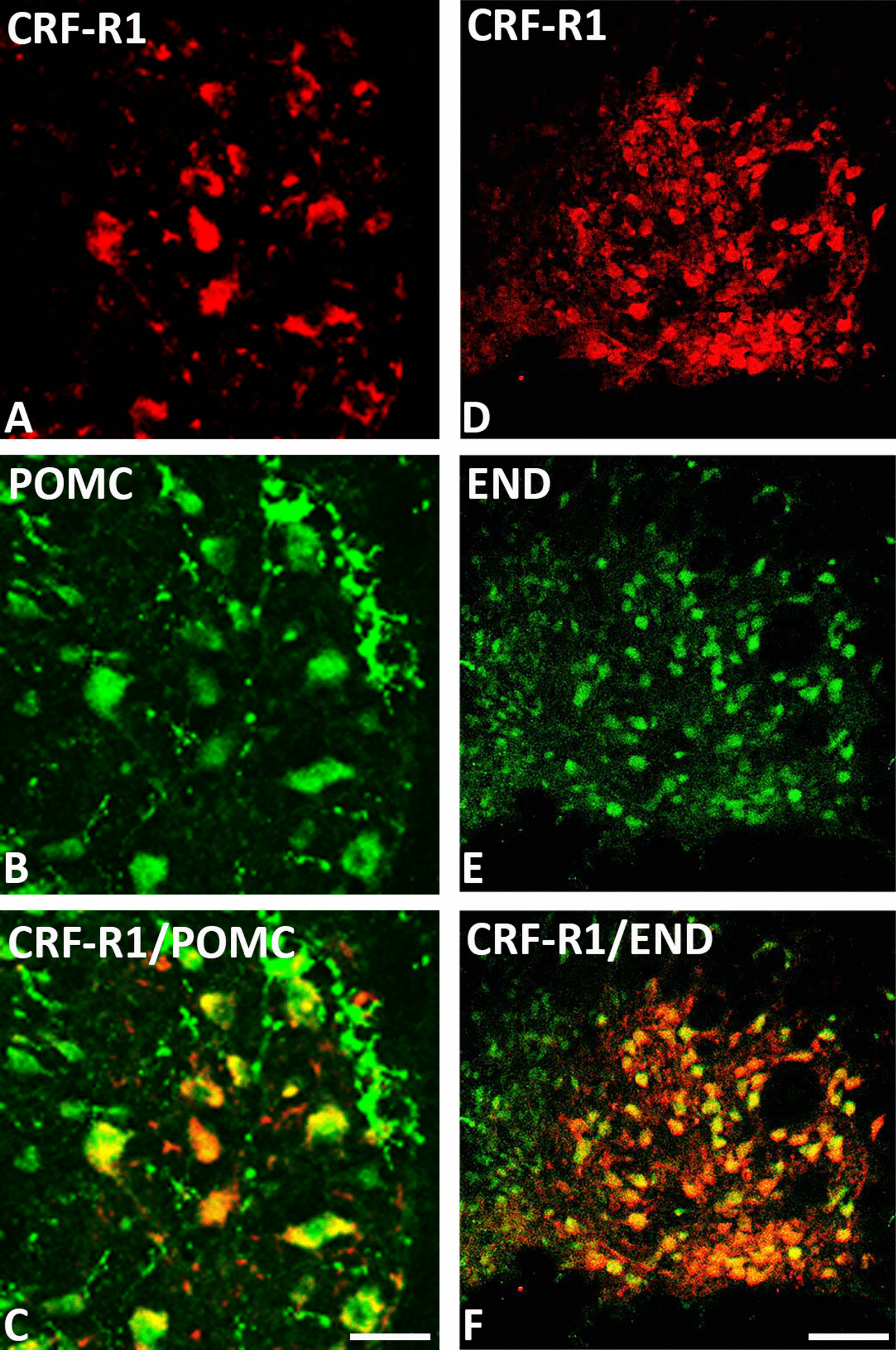
Double-immunofluorescence staining of CRF-R1 (*red fluorescence*) **A**, **D** with proopiomelanocortin (POMC) **B** or β-endorphin (END) **E** (*green fluorescence*) in the Locus coeruleus (LC) of the rat brain. **A**–**F** Double-immunofluorescence staining of coronal sections of the rat brain with hind paw inflammation showing co-expression of CRF-R1 with POMC **C** or END **F** within LC. Some neurons express CRF-R1, POMC or END only. Bar = 20 µm for **A**–**C**. Bar = 40 μm for (**D**–**F**)

Our double-immunofluorescence confocal microscopy of rat brain sections revealed that predominantly CRF-R1 are expressed in opioid precursor POMC—as well as opioid peptide (3E7^+^)—positive neurons within multiple pain-relevant brain areas, such as the thalamus, hypothalamus, periaquaductal grey and locus coeruleus (Figs. 4, 5, 6 and 7). Indeed, in frontal brain sections of FCA-treated rats, CRF-R1 immunoreactivity was strongly visible compared to CRF-R2 immunoreactivity (Figs. 4, 5). Moreover, double-immunofluorescence confocal microscopy confirmed the co-expression of CRF-R1 with the opioid peptide precursor POMC and with its end product ß-endorphin in the paraventricular nucleus (PVN), the median eminence and the supraoptic area (SOA) of the hypothalamus in the rat brain. In addition, we assessed the co-expression of CRF-R1 and vasopressin that occupied the same hypothalamic sub-region (Figs. 4, 7). Interestingly, CRF-R1 tended to reside in the same hypothalamic region as vasopressin, yet there was no (Fig. 4D-F) or rare (Fig. 7G-I) overlap of the two cell groups. In parallel, there was no (Fig. 4G-I) or scarce (Fig. 7J-L) co-localization of CRF-R2 with the opioid peptide precursor POMC. In the thalamus and periaqueductal grey regions, CRF-R1-positive neurons co-expressed the opioid peptide precursor POMC (Fig. 6). Some neurons within the thalamus and the periaqueductal grey were immunoreactive for opioid peptides only (Fig.  6). Within the locus coeruleus, the majority of CRF-R1 immunoreactive neurons expressed POMC as well as ß-endorphin, but few neurons showed immunoreactivity of CRF-R1, POMC or ß-endorphin alone (Fig. 8).

**Fig. 9:**
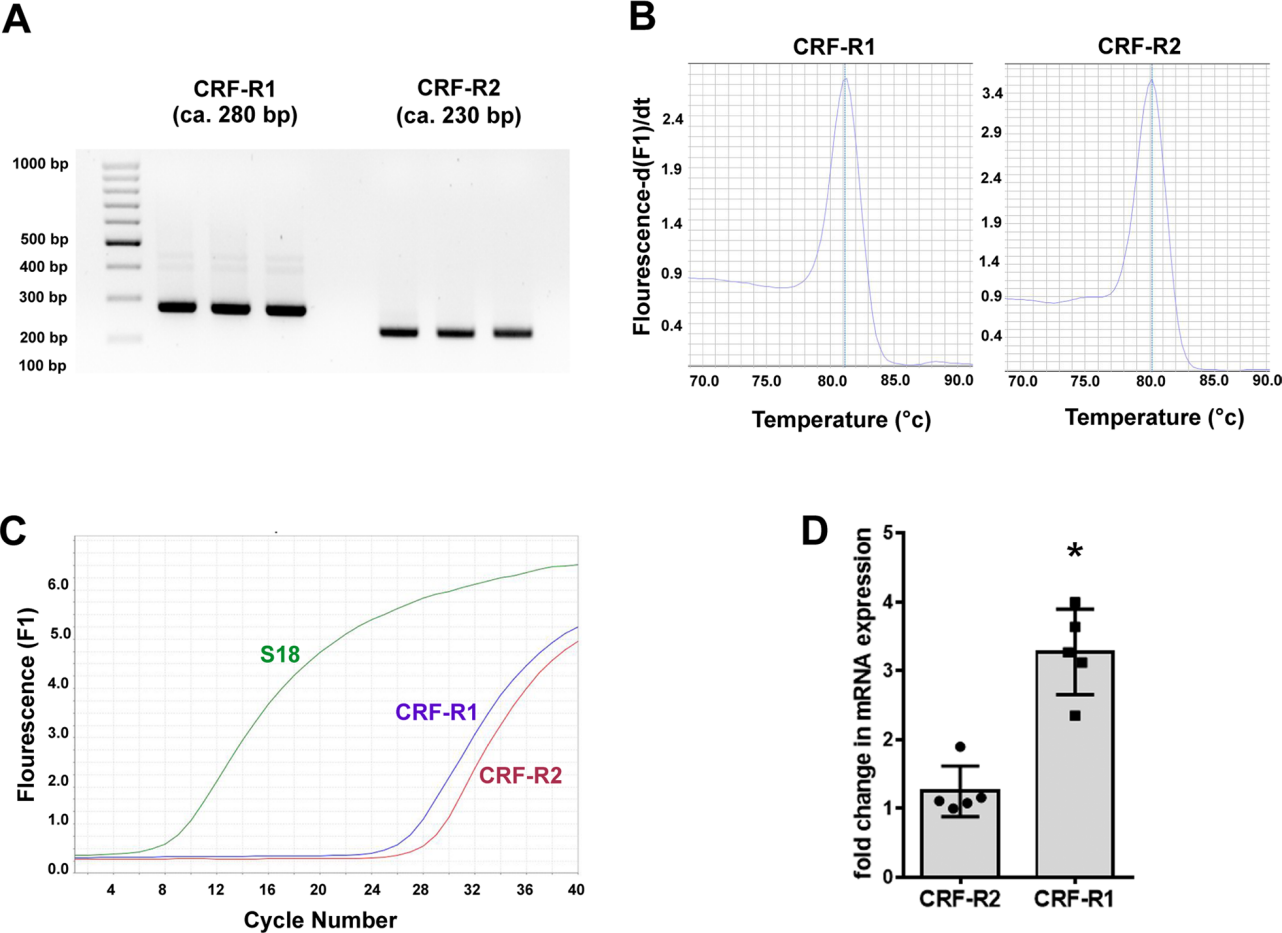
CRF-R1 and CRF-R2 mRNA expression in the hypothalamus region of the rat with 4 day FCA-induced hind paw inflammation. **A** RNA extraction from the rat hypothalamus, implementation of conventional PCR using specific primer pairs for CRF-R1 and CRF-R2, and subsequent visualization on a 2% agarose gel provided specific PCR products for the expression of CRF-R1 (280 bp) and CRF-R2 (230 bp) mRNA. **B** Shows the DNA melting profiles of the CRF-R1 (right) and CRF-R2 (left) specific primer pairs. **C**, **D** Quantification of CRF-R1 and CRF-R2 mRNA using Taqman® Real-Time PCR in the hypothalamus region of the rat brain. **C** The amplification profiles of the 18S- and CRF-R1- and CRF-R2-specific cDNA of rat hypothalamus. **D** The column graph representing % CRF-R1 mRNA expression relative to the expression of CRF-R2 mRNA. Note that CRF-R1 expression is more than threefold higher than that of CRF-R2 (experiments were done in duplicate from *n* = 5 rats, *P < 0.05, two-tailed independent Student t-test)

Since the hypothalamus plays a central role in the coordination of autonomic and behavioral aspects of the response to painful stimuli [26], we examined the differential expression of CRF-R1 and CRF-R2 mRNA in the hypothalamus as a representative brain area. Using a highly specific primer pair, both CRF-R1 and CRF-R2 mRNA were detectable in the rat hypothalamus (Fig. 9). Intriguingly, the CRF-R1 expression was predominant over CRF-R2, since CRF-R1 mRNA levels were significantly higher than that of CRF-R2 (*P* < 0.05, two-tailed independent Student t-test) (Fig. 9) (Additional file 1: Fig. S1).

## Discussion

In the present study, we aimed to determine the role of brain CRF receptor subtypes in a rat model of inflammatory pain. Here, we demonstrated that i.c.v. CRF elicited potent, dose-dependent antinociceptive effects in inflammatory pain which to a great extent was antagonized by i.c.v. CRF-R1 selective antagonist NBI 27914 and only partially by the CRF-R2 selective antagonist K41498. Consistently, i.c.v. treatment with the CRF-R1 agonist stressin I produced superior antinociceptive effects in comparison to the CRF-R2 agonist Ucn-2. I.c.v. injected opioid antagonist naloxone significantly reversed the CRF—as well as CRF-R1 agonist-mediated pain inhibition. Confocal fluorescent microscopy identified mostly CRF-R1-positive neurons expressing opioid peptides in pain-relevant brain areas with only scarce presence of CRF-R2-positive neurons. Taken together, these findings suggest a superior involvement of neuronal CRF-R1 versus CRF-R2 in the central modulation of inflammatory pain through endogenous opioids.

In contrast, intrathecal CRF or selective CRF-R2 agonists induced dose-dependent antinociception in animals with inflammatory pain which could be fully blocked by intrathecal CRF-R2 antagonist as well as the opioid receptor antagonist naloxone [14]. On the other hand, intrathecal CRF-R1 agonist effects were neglected which was in line with the sparse CRF-R1 expression in the spinal cord and the co-localization of spinal CRF-R2 receptors with enkephalinergic inhibitory interneurons [14]. We have now extended these studies by examining the specific role of each CRF receptor subtype on centrally administered CRF-induced antinociception in a rat model of inflammatory pain. It is well accepted that CRF-R1 is predominantly expressed in the brain and that i.c.v.-administered CRF reaches high enough concentrations to evoke c-Fos expression in pain-relevant brain regions such as the thalamus, hypothalamus, locus coeruleus, and periaqueductal gray, as previously described [4, 27].

Consistent with a previous study [4], we found that i.c.v.-administered CRF induces antinociception during inflammatory pain. Moreover, the central co-administration of CRF with the selective CRF-R1 antagonist NBI 27914 grossly abolished the CRF-induced antinociception, whereas the CRF-R2 receptor selective antagonist K41498 did so only partially. Further substantiating these findings, i.c.v. application of the CRF-R1 selective agonist stressin I greatly increased nociceptive thresholds during inflammatory pain, whereas the CRF-R2 selective agonist urocortin-2 did so to a much lesser extent. This is in line with the central role of brain CRF receptors during stress-induced analgesia [28] and sustained painful stimuli [29] as well as the crucial role of CRF-R1 receptors in the brain during contextually conditioned fear [30]. However, our results are in contrast to recent studies that mainly focused on the pronociceptive role of CRF receptors within the central nucleus of the amygdala [31, 32]. This discrepancy can be explained by differences in the potency, efficacy and availability of corresponding receptor subtypes within distinct pain-relevant brain areas as well as by the differences in the animal models of pain. In agreement with our observation, i.c.v. CRF-induced increased visceromotor sensitivity was blocked by a centrally administered non-selective CRF receptor antagonist [17] or a systemically administered CRF-R1-selective antagonist [16]. To examine whether the effects of CRF as well as CRF-R1 agonist are opioid-mediated, we examined the concomitant i.c.v. injection of the opioid receptor antagonist naloxone with CRF or CRF-R1 agonist, respectively. Indeed, we found that the antinociceptive effect of i.c.v. CRF or CRF-R1-selective agonist was dose-dependently reversed by naloxone suggesting that anti-nociception of i.c.v. CRF or CRF-R1 agonist NBI 27914 is mediated through endogenous opioid peptides within the brain. At present, there is only one study that actually showed an endogenous opioid peptide release from neurons within the brain of animals with inflammatory pain [33]. Our findings are in contrast to the study by Yarushkina et al. [34] in which i.c.v. applied CRF-induced antinociceptive effects apparently by a non-opioid mechanism via the activation of the hypothalamic–pituitary–adrenal stress axis [34]. I.c.v. administered CRF, however, was injected at a 20-fold higher dose in an animal model of electrically evoked pain, making a direct comparison problematic.

Supporting the present behavioral findings, double-immunofluorescence confocal microscopy showed predominant expression of CRF-R1 and less of CRF-R2 in pain-relevant brain areas that co-localized with the opioid peptide precursor POMC or its endproduct ß-endorphin. Our CRF-R1 immunoreactivity was clearly detectable in brain areas using a specific antibody against CRF-R1 (Figs. 4, 5, 6 and 7) similar to previous findings of known CRF-R1 expression within the hypothalamus using CRF-R1-GFP transgenic mouse brain [35, 36] which confirms the specificity of the antibody. Additionally, in our previous study in the rat spinal cord [14], western blot analysis revealed specific CRF-R1 and CRF-R2 immunoreactive bands at the expected molecular weight of 38 and 56 kDa, respectively.

It is well established that the thalamus, hypothalamus, locus coeruleus, and periaqueductal gray are the main brain areas involved in pain modulation as reviewed by Mills et al., [37]. Indeed, brain imaging studies [38] and neuropsychological investigations [37] have identified brain areas of the hypothalamus, thalamus, periaqueductal grey and locus coeruleus as specific areas for pain modulation [39]. Therefore, in our immunohistochemical experiments, we focused on these known brain areas and demonstrated high abundance of CRF-R1 receptors in the paraventricular nucleus (PVN), similar to the previously reported CRF-R1 expression within the hypothalamus using CRF-R1-GFP transgenic mouse brain [35, 36, 40], in the median eminence of the hypothalamus, the reticular thalamic nucleus [41] and the periaqueductal area as well as the supraoptic area and locus coeruleus [35]. These observations are in line with previous reports suggesting that CRF-R1 is expressed in the rodent brain at higher levels compared to CRF-R2 [42, 43] and has a greater binding affinity to CRF [44]. Since the hypothalamus plays a central role in the coordination of autonomic and behavioral aspects of the response to painful stimuli [26], we focused our quantitative RT-PCR experiments on the known hypothalamic brain areas. Intriguingly, our quantitative real-time PCR also showed a more than threefold expression of CRF-R1 compared to CRF-R2 in the rat hypothalamus.

In these pain-relevant brain areas, we found abundant co-localization of CRF-R1 with POMC or its endproduct ß-endorphin [4] but not of CRF-R2 receptors and not of other neuropeptides such as vasopressin [36]. Taken together, these findings taken from behavioral, morphological as well as PCR experiments suggest that i.c.v. CRF or CRF-R1 agonists inhibit somatic inflammatory pain predominantly through CRF-R1 receptors located in pain-relevant areas, finally resulting in endogenous opioid-mediated pain inhibition.

Several limitations should be addressed in order to stimulate further study in the future. First, our experiments evaluated i.c.v. effects of CRF agonist during inflammatory pain only following mechanical nociceptive stimulation. Second, we have evaluated only short-lasting acute effects of i.c.v. CRF in our behavioral experiments. Finally, we have examined the i.c.v. effects of CRF agonists only after i.c.v. application without specifically targeting certain brain areas.

## Conclusion

In summary, we have demonstrated in an animal model of persistent inflammatory pain that the neuronal CRF-R1 within the rat brain plays an important role in somatic pain modulation through endogenous opioids. Indeed, i.c.v. CRF receptor agonists elicited potent antinociceptive effects in inflammatory hyperalgesia, which was antagonized mostly by i.c.v. CRF-R1 yet much less by CRF-R2 antagonist. Consistently, the analgesic effect of i.c.v. CRF-R1 agonist was superior compared to that produced by the CRF-R2 agonist, indicating superior involvement of brain CRF-R1 receptors over CRF-R2. Moreover, i.c.v. opioid receptor antagonist naloxone dose-dependently reversed either CRF’s or CRF-R1 agonist’s antinociceptive effects. Consistently, we have identified a co-localization of opioid peptides with predominant CRF-R1 in neurons within pain-relevant brain areas. The data presented in our study identify CRF-R1 as the most effective and potent CRF receptor subtype within brain areas in inflammatory pain modulation and strengthen the evidence of the involvement of the opioid system.

## Supplementary Information

**Additional file 1: Fig. S1**: Showing the original gel of the cDNA nucleotide bands of CRF-R1 (280 bp) and CRF-R2 (230 bp) in Fig. 9.

## Data Availability

The datasets used and/or analyzed during the current study are available from the corresponding author (Shaaban.mousa@charite.de) on reasonable request. The authors will take responsible for maintaining availability.

## Abbreviations

CRF: Corticotropin-releasing factor
CRF-R1: CRF receptors subtypes 1
CRF-R2: CRF receptors subtypes 2
FCA: Freund’s complete adjuvant
i.c.v.: Intracerebroventricular
PPT: Paw pressure thresholds
PCR: Polymerase chain reaction
