# Supplementary figures and images for: Superior control of inflammatory pain by corticotropin-releasing factor receptor 1 via opioid peptides in distinct pain-relevant brain areas
Source: J Neuroinflammation. 2022 Jun 15;19:148. doi: 10.1186/s12974-022-02498-8 (PMC9199204; doi:10.1186/s12974-022-02498-8)

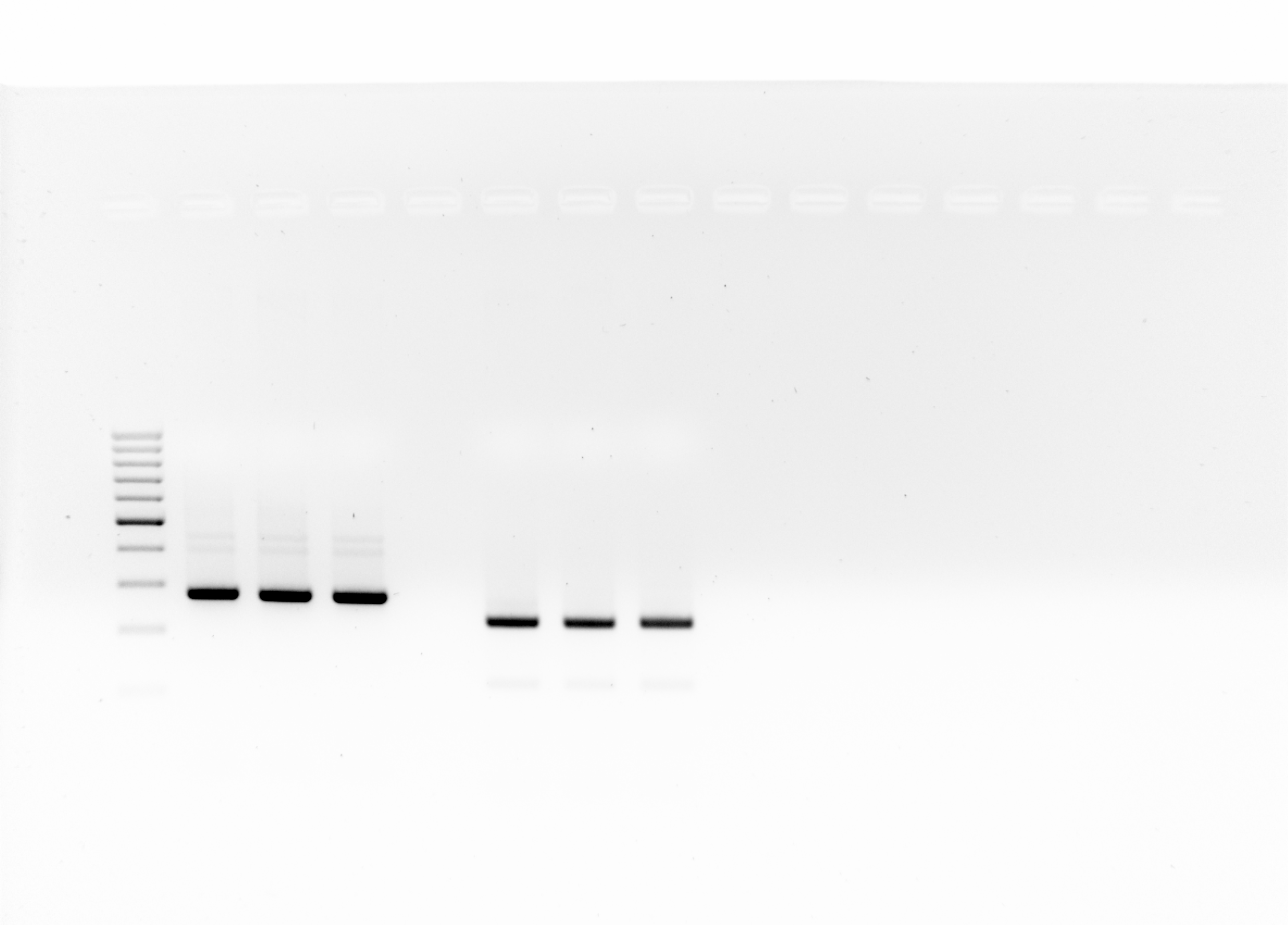

Supplement: Supplementary file 1 — Additional file 1: Fig. S1: Showing the original gel of the cDNA nucleotide bands of CRF-R1 (280 bp) and CRF-R2 (230 bp) in Fig. 9. [file 12974_2022_2498_MOESM1_ESM.tif]
